# Matched-Pair Analysis of Survival in the Patients with Oropharyngeal Squamous Cell Carcinoma Treated with Radiotherapy or Concurrent Chemoradiotherapy Versus Surgical-Based Treatment

**DOI:** 10.3390/cancers17233779

**Published:** 2025-11-26

**Authors:** Patricia García-Cabo, Daniel Pedregal-Mallo, Pilar Blay, Maria Ángeles de la Rúa, Maria Pilar Solis-Herndez, Sonia Blanco, José Luis Llorente, Juan P. Rodrigo, Fernando López

**Affiliations:** 1 Department of Otorhinolaryngology, Head and Neck Surgery, Hospital Universitario Central de Asturias (HUCA), 33011 Oviedo, Spain; patricia.garcia-cabo@sespa.es (P.G.-C.); daniel.pedregal@sespa.es (D.P.-M.); jllorentep@uniovi.es (J.L.L.); jprodrigo@uniovi.es (J.P.R.); 2 Department of Medical Oncology, Hospital Universitario Central de Asturias (HUCA), 33011 Oviedo, Spain; pilar.blay@sespa.es (P.B.); mariadelpilar.solis@sespa.es (M.P.S.-H.); 3 Department of Radiation Oncology, Hospital Universitario Central de Asturias (HUCA), 33011 Oviedo, Spain; angeles.delarua@sespa.es (M.Á.d.l.R.); sonia.blanco@sespa.es (S.B.); 4 Instituto de Investigación Sanitaria del Principado de Asturias (ISPA), Instituto Universitario de Oncología del Principado de Asturias (IUOPA), University of Oviedo, CIBERONC-ISCIII, 33011 Oviedo, Spain

**Keywords:** oropharyngeal squamous cell carcinoma, chemoradiotherapy, surgery, survival, matched-pair analysis

## Abstract

Background: This study compared survival outcomes between upfront surgery +/−radiotherapy (RT) and definitive RT with or without chemotherapy (RT/CRT) in HPV-negative oropharyngeal squamous cell carcinoma (OPSCC). Methods: A retrospective matched-pair analysis included 150 patients treated between 1990 and 2020. Fifty (RT/CRT) cases (2009–2020) were matched 2:1 to 100 surgical cases (1990–2009) by sex, subsite, T/N classification, and AJCC 8th stage. Sixty-six percent of surgically treated patients received adjuvant RT. Only HPV-negative tumors confirmed by p16 and HPV DNA testing were analyzed. Survival was assessed with Kaplan–Meier and Cox regression. Results: RT/CRT achieved significantly higher 5-year overall survival (59% vs. 28%) and disease-specific survival (69% vs. 45%) compared with surgery. Matched-pair analysis showed increased overall mortality (HR 1.91; *p* = 0.008) and disease-specific mortality (HR 2.05; *p* = 0.022) in surgical patients. In stage III disease, RT/CRT provided markedly superior DSS (87% vs. 42%). No significant differences were found in local control or recurrence-free survival, but metastasis-free survival was significantly worse in the surgical cohort, and the incidence of second primary tumors was significantly lower in the RT/CRT group. Conclusions: These findings indicate that RT/CRT provides superior survival outcomes and better control of distant metastasis and second primary tumors compared with surgery in HPV-negative OPSCC, particularly in stage III disease. RT/CRT should be considered an effective organ-preserving option for this population.

## Simple Summary

The optimal treatment for oropharyngeal squamous cell carcinoma (OPSCC) remains controversial, particularly in HPV-negative tumors, which have a poorer prognosis than HPV-positive disease. This study compared survival outcomes in patients treated with upfront surgery versus definitive radiotherapy or chemoradiotherapy (RT/CRT) using a matched-pair analysis of 150 cases. All tumors were confirmed HPV-negative. We found that RT/CRT achieved significantly better overall and disease-specific survival, especially in stage III disease, and reduced the risk of distant metastases and second primary tumors compared with surgery. These findings support RT/CRT as an effective organ-preserving treatment option in HPV-negative OPSCC, providing comparable or superior oncologic outcomes to surgery while avoiding the morbidity of extensive open procedures.

## 1. Introduction

The 5-year overall survival (OS) of oropharyngeal squamous cell carcinoma (OPSCC) varies substantially depending on HPV status, tumor stage, and clinical risk factors. In unselected series, the 5-year OS lies approximately between 45% and 57%. HPV-positive tumors show substantially better outcomes (around 65–79%), whereas HPV-negative cases present markedly poorer survival (28–35%) [1,2,3,4]. In the past, 75–85% of OPSCC were attributed to tobacco and alcohol consumption. In more recent decades, however, an increasing proportion of OPSCCs are linked to human papillomavirus (HPV) infection. The global prevalence of HPV-attributable OPSCC is region-dependent but has been estimated in many series to approximate 30–35% [5,6].

HPV-positive OPSCC therefore has emerged as a distinct clinical entity, characterized by improved prognosis, better response to therapy, and significantly higher survival rates in contrast to HPV-negative disease. Accordingly, HPV status now features prominently as a stratification factor in staging and therapeutic decision-making, with separate considerations applied to HPV-positive tumors [6,7].

Over time, treatment paradigms for OPSCC have evolved substantially. Until the 1990s, open surgical resection (with subsequent adjuvant radiotherapy (RT)) was the mainstay approach. Given the morbidity associated with extensive surgical procedures, concurrent chemoradiotherapy (CRT) became broadly adopted as standard of care for many patients. While CRT has demonstrated efficacy, it also carries considerable long-term toxicity and functional impairment, particularly in swallowing and speech [8,9].

The advent of minimally invasive techniques—such as transoral laser microsurgery (TLM) and transoral robotic surgery (TORS)—has transformed the surgical approach. These techniques have afforded oncologic outcomes comparable to conventional surgery but with better functional preservation and lower treatment-related morbidity [10,11,12]. This is particularly relevant in HPV-positive patients, who are often younger and have longer life expectancy, so that quality of life becomes a key issue [6]. As a result, de-intensification treatment strategies using minimally invasive surgery have received increasing attention, particularly in HPV-associated early-stage disease, with the objective of reducing therapeutic burden while maintaining oncologic efficacy [1,13].

In locally advanced OPSCC, major international guidelines consistently identify surgery followed by postoperative RT with or without concurrent chemotherapy, and definitive cisplatin-based CRT, as standard treatment options. Both the NCI PDQ^®^ [14] and the EHNS–ESMO–ESTRO [10] guidelines emphasize that treatment selection must be individualized according to pathological risk factors, anticipated functional impact, and patient comorbidity. Altered-fractionation RT (accelerated or hyperfractionated) remains an evidence-based alternative when chemotherapy is contraindicated. For resectable tumors whose removal would entail major functional compromise, non-surgical CRT is often preferred, whereas postoperative CRT is mandatory in the presence of positive margins or extracapsular extension.

Recent evidence suggests improved outcomes compared with historical cohorts. A meta-analysis of primary surgery in HPV-negative OPSCC reported a pooled 5-year overall survival (OS) of 72% for surgery followed by risk-adapted RT, and up to 82% in carefully selected patients undergoing transoral approaches, underscoring the potential benefit of appropriately selected surgical management in this subgroup [15]. By contrast, early-stage OPSCC (cT1–T2N0M0) is typically managed with single-modality therapy—either radiotherapy alone or transoral surgery with ipsilateral neck dissection. Most patients with stage III–IV disease, however, require combined-modality treatment, including definitive CRT or surgical resection with oropharyngeal reconstruction followed by adjuvant therapy according to pathological risk stratification. When different strategies provide comparable oncologic outcomes, patient preference and quality-of-life considerations should guide the therapeutic decision. Given the increasing complexity of management, OPSCC should ideally be treated in high-volume specialized centers within multidisciplinary tumor boards that integrate clinical, pathological, and molecular data to optimize selection and timing of therapy [5].

In this context, we conducted a matched-pair analysis comparing the two principal treatment modalities—surgery and CRT—in patients with HPV-negative OPSCC, evaluating their impact on locoregional control, disease-specific survival, and overall survival. The specific contribution of this study is to provide contemporary, risk-adjusted evidence on the comparative effectiveness of upfront surgery versus definitive chemoradiotherapy in HPV-negative OPSCC, a subgroup with historically poor outcomes and for which robust matched analyses remain limited. Existing benchmarks for HPV-negative disease are highly heterogeneous, often derived from population-based registries or historical surgical and CRT cohorts that lack HPV stratification, uniform staging, or treatment comparability. Reported survival outcomes range widely—typically between 28% and 35% in unselected series [1,2,3,4] and up to 70–82% in optimized surgical cohorts—reflecting substantial differences in case mix, treatment era, and methodological rigor [13]. By focusing exclusively on HPV-negative tumors and constructing meticulously matched groups according to T/N classification, subsite, and stage, our study provides a controlled comparison aligned with—and directly contrasting—these benchmark datasets. This design allows us to determine whether the survival advantage suggested in modern surgical series persists when compared to contemporary CRT outcomes under balanced conditions, thereby refining current benchmark expectations and supporting more informed therapeutic decision-making in this high-risk population.

## 2. Materials and Methods

### 2.1. Patients

A retrospective review was conducted of 150 patients diagnosed with OPSCC and treated at the Hospital Universitario Central de Asturias between 1990 and 2020. The study received approval from the Institutional Review Board of our center. Owing to its retrospective and observational design, informed consent for participation was not required.

The study population was identified first, after which eligibility criteria were applied, HPV-negative status was confirmed, and matched groups were constructed before analyzing treatment outcomes.

The oropharyngeal primary tumor sites include the soft palate, tonsillar pillar, palatine tonsil, base (posterior one-third) of the tongue (BOT), vallecula, and oropharyngeal walls. For this study only patients with tumors located in the base of the tongue or the palatine tonsil were included.

Two homogeneous groups were selected: 50 previously untreated patients with OPSCC who received concurrent CRT between 2009 and 2020, and a control group of 100 patients treated with up-front surgery between 1990 and 2009. Patients who did not complete treatment with RT or CRT were excluded. Both groups were statistically comparable in terms of disease stage (TNM, 8th edition), tumor subsite, sex, and HPV status. None of the patients had distant metastases at diagnosis. Minimum follow-up was 24 months or until death.

HPV status was determined using p16 immunohistochemistry. Tumors showing p16 positivity (defined as ≥70% of cells with strong nuclear and cytoplasmic staining) underwent additional testing for high-risk HPV DNA through GP5+/6+ PCR, followed by enzyme immunoassay detection covering 14 high-risk HPV types. Genotyping of HPV-positive cases was performed using a bead-based Luminex array. In situ hybridization was also carried out on all tumors using biotinylated probes for HPV types 16, 18, 31, 33, 35, 39, 45, 51, 52, 56, 58, 59, and 68 (Y1443; Dako Cytomation—Glostrup, Denmark) on 3-µm formalin-fixed paraffin-embedded TMA sections, as previously described [9,10,11]. To maintain a uniform study population, only HPV-negative patients were included.

After defining both cohorts and completing the matching process, the specific management protocols used for non-surgical therapy and up-front surgery during their respective periods are described below to clarify how each group was treated.

Non-surgical therapy—comprising intensity-modulated radiation therapy (IMRT) with or without chemotherapy—was delivered to patients with histologically confirmed, previously untreated OPSCC who presented with advanced disease (stage III or IV), had medical contraindications to surgery, or declined surgical management despite being in early stages (I or II). Staging workup included a physical examination and contrast-enhanced computed tomography (CE-CT), or positron emission tomography combined with CE-CT. Definitive radiotherapy was delivered with conventional fractionation, once daily, five days per week, using 2 Gy per fraction. The initial large-field irradiation encompassed the primary tumor, involved lymph nodes, and nodal regions at risk. Doses to these large fields ranged from 46 to 50 Gy, and the total dose to gross disease and involved nodes reached 70 Gy. For patients with advanced disease and no contraindications to systemic therapy, chemoradiotherapy (CRT) was administered, with drug regimens and schedules individualized according to medical history and treatment tolerance. Twelve weeks after completing CRT, tumor response was assessed separately for the primary tumor and regional lymph nodes. Follow-up evaluation included a neck PET-CT and flexible laryngoscopy, with biopsy performed for any suspected persistent primary lesion. Patients with residual nodal disease were considered candidates for radical or modified radical neck dissection, and those with biopsy-confirmed persistent primary disease underwent salvage surgery whenever feasible.

Matched-pair controls were selected according to sex, T classification, N classification, and tumor subsite from patients treated with up-front surgery between 1990 and 2009. This approach represented the primary treatment protocol implemented at our institution during the specified period. Surgery was offered to medically fit patients after comprehensive preoperative evaluation. The indication for surgery was based on detailed clinical assessment and cervical CE-CT imaging. Patients with early-stage tumors and good exposure underwent intraoral resections using either monopolar electrosurgery or CO_2_ laser microsurgery. For advanced tumors or limited exposure, transmandibular or transpharyngeal approaches were used. Clinically N0 patients underwent unilateral or bilateral selective neck dissection. In N+ patients, ipsilateral or bilateral modified or radical neck dissection was performed. Postoperative RT was administered in cases of T3-T4 tumors, pN2–N3 nodal disease, extranodal extension, or positive margins. RT was delivered using conventional fractionation (2.0 Gy per fraction, five fractions per week), with a total dose ranging from 56 to 64 Gy depending on the risk factors. Patients with positive resection margins received a total dose of 66–70 Gy to areas of residual disease. It should be noted that, during the period 1990–2009, adjuvant chemoradiotherapy was not part of the standard protocol at our institution, which explains why none of the surgically treated patients received concurrent therapy.”

### 2.2. Statistical Analysis

Statistical analyses were carried out using IBM SPSS version 26.0. Categorical variables were analyzed with the Chi-square test. Survival outcomes were estimated using the Kaplan–Meier approach, and comparisons between survival curves were performed with the log-rank test. Multivariable evaluation relied on the Cox proportional hazards model. The matching design was incorporated into the Cox models through a dedicated matching variable reflecting sex, tumor site, T category, N category, and stage. All statistical tests were two-sided, and a *p*-value < 0.05 was deemed significant.

## 3. Results

### 3.1. Characteristics of the Patient Population

Baseline demographic and tumor features are presented in Table 1. The median age was 59 years (range: 41–81), and all individuals included were men. As matching was applied for sex, tumor subsite, T and N categories, and AJCC stage, no significant differences were detected between treatment groups for these variables. Age distribution and histologic grade were also comparable between groups (*p* > 0.05). In contrast, alcohol use showed a significant imbalance (*p* = 0.001), with a greater proportion of heavy drinkers in the surgery group. The median follow-up period was 31 months (mean: 49 months; range: 1–393 months).

Among the 50 patients treated with non-surgical management, CRT was administered in 39 cases. Cisplatin (100 mg/m^2^ on days 1, 22, and 43) was the most frequently used regimen (*n* = 30). One patient received weekly carboplatin (AUC = 2), and eight patients were treated with cetuximab (loading dose 400 mg/m^2^ followed by 250 mg/m^2^ weekly). Among the 50 patients treated with CRT, 26 (52%) developed either persistent or recurrent disease. Of these, recurrence was classified as local in 8 patients (30.8%), regional in 3 (11.5%), locoregional in 5 (19.2%), and distant in 3 (11.5%). Additionally, 6 patients (12%) developed a second primary tumor, most commonly in the lung (4 cases; 66.7%), followed by the esophagus (2 cases; 33.3%). The mean time to recurrence was 18 months (range, 4–130 months). Salvage surgery was pursued in 12 patients (46.2%), palliative chemotherapy in 3 (11.5%), and the remaining 11 (42.3%) received symptomatic management only. At the time of analysis, 10 of the patients with persistence or recurrence (38.5%) were alive and disease-free.

In the surgical cohort (*n* = 100), 20 patients (20%) underwent transoral resection, while 80 patients (80%) received open surgical approaches: 49 via a transmandibular approach and 31 via a transpharyngeal approach. Neck dissection was performed in nearly all cases (99%), and 66 patients (66%) received postoperative RT. In these cohort (*n* = 100), 60 patients (60%) developed either persistent or recurrent disease. Recurrences were distributed as follows: 19 local (31.7%), 2 regional (3.3%), 16 locoregional (26.7%), and 19 distant (31.7%). Four patients (6.7%) developed a second primary tumor, all located in the lung. The mean interval to recurrence was 24 months (range, 4–371 months). Salvage surgery was feasible in only 4 cases (6.7%), whereas the remaining 56 (93.3%) received palliative management, as reirradiation was not considered. At the time of evaluation, just 5 of the patients with persistence or recurrence (8.3%) were alive and disease-free.

### 3.2. Survival Outcomes

**Figure 1:**
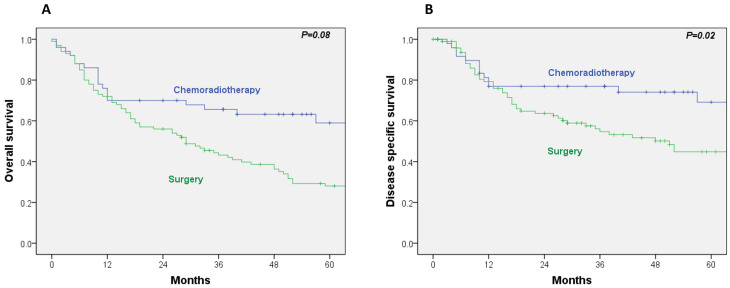
(**A**) Overall survival by treatment; (**B**) disease-specific survival by treatment.

The 5-year OS and disease-specific survival (DSS) rates in the RT/CRT group were 59% and 69%, respectively. In comparison, the surgical group exhibited 5-year OS and DSS rates of 28% and 45%. Univariate Cox regression analysis revealed a significantly lower DSS in the surgical group (HR, 2.06; 95% CI, 1.11–3.82; *p* = 0.02), while the difference in OS did not reach statistical significance (HR, 1.98; 95% CI, 1.18–3.07; *p* = 0.08) (Figure 1A,B).

**Figure 2:**
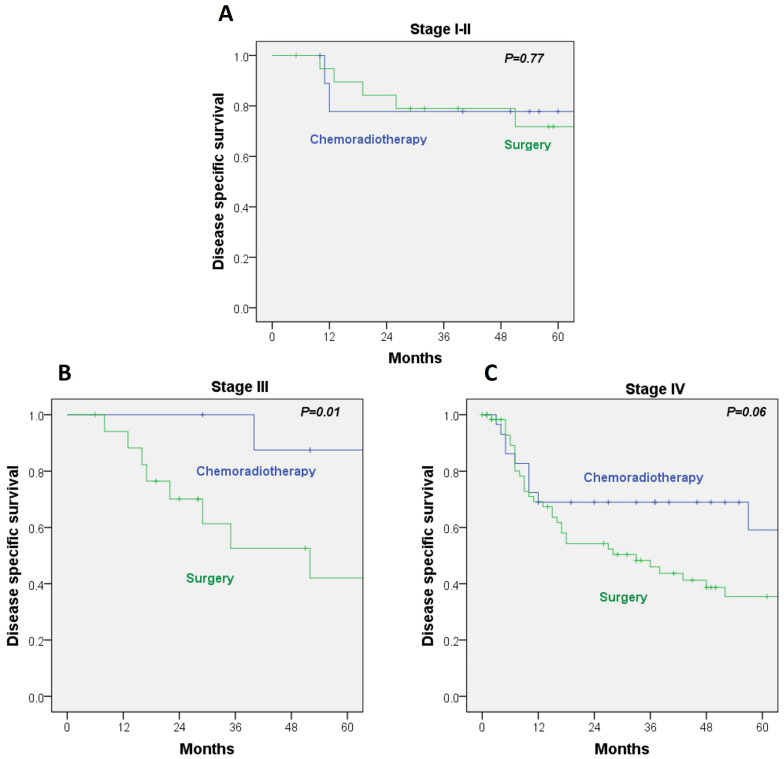
(**A**) Disease-specific survival by treatment in stage I–II; (**B**) disease-specific survival by treatment in stage III; (**C**) disease-specific survival by treatment in stage IV.

When DSS was analyzed within AJCC stages, no significant differences emerged between treatment modalities for stage I–II disease (HR, 1.26; 95% CI, 0.25–6.32; *p* = 0.77). In contrast, among stage III patients, DSS was significantly better in the RT/CRT group, with a 5-year DSS of 87% versus 42% in the surgery cohort (HR, 1.55; 95% CI, 1.11–2.16; *p* = 0.01). A comparable pattern was noted in stage IV disease, although the difference did not reach statistical significance (HR, 7.08; 95% CI, 0.87–57.4; *p* = 0.06) (Figure 2A–C). These findings indicate that the survival advantage of RT/CRT is not consistent across all stages and is statistically significant only in stage III. No benefit was observed for early-stage disease, and the apparent trend in stage IV did not reach significance.

**Figure 3:**
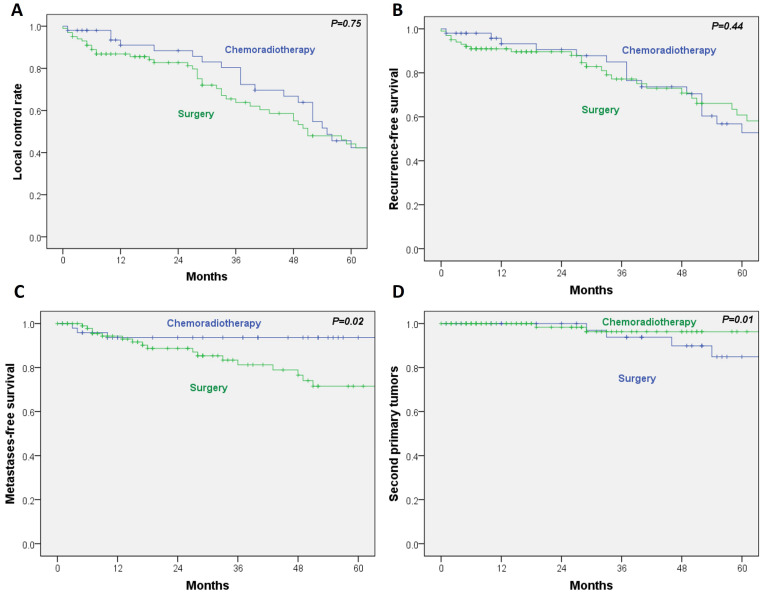
(**A**) Local control; (**B**) recurrence-free survival; (**C**) metastasis-free survival; and (**D**) second primary tumor rate, by treatment.

For the secondary outcomes, no statistically significant differences were observed between RT/CRT and surgery in local control (HR, 0.93; 95% CI, 0.59–1.45; *p* = 0.75) or recurrence-free survival (HR, 0.81; 95% CI, 0.48–1.37; *p* = 0.44). In contrast, metastasis-free survival was significantly poorer in the surgical group (HR, 3.5; 95% CI, 1.03–11.9; *p* = 0.02). Additionally, the incidence of second primary tumors was significantly lower in the RT/CRT cohort (HR, 0.12; 95% CI, 0.02–0.62; *p* = 0.01) (Figure 3A–D).

In the multivariable logistic regression analysis including age, alcohol and tobacco use, T-classification, N-classification, and histologic grade, only T- (OR, 2.00; 95% CI, 1.06–3.76; *p* = 0.03) and alcohol use (OR, 1.68; 95% CI, 1.17–2.42; *p* = 0.05) were independently associated with DSS. For OS, both T-classification (OR, 1.75; 95% CI, 1.06–2.88; *p* = 0.027) and N-classification (OR, 4.51; 95% CI, 1.31–15.46; *p* = 0.017) emerged as significant predictors.

Because 66% of patients in the surgical cohort received adjuvant radiotherapy, this group predominantly represents a combined-modality approach (surgery + RT), which reflected the standard practice in our institution during 1990–2009. The stage-stratified analyses (Figure 2) help contextualize this effect, demonstrating that differences between treatment modalities mainly arise in stage III and IV disease, while no significant disparities were observed in early-stage (I–II) tumors.

### 3.3. Matched Analysis of Survival

Matched Cox regression analysis demonstrated that, compared to RT/CRT, upfront surgery was associated with a significantly increased risk of death (HR, 1.91; 95% CI, 1.18–3.08; *p* = 0.008) and disease-specific mortality (HR, 2.05; 95% CI, 1.11–3.81; *p* = 0.022).

## 4. Discussion

Historically, the surgical management of OPSCC beyond early-stage tonsillar lesions required extensive open approaches—such as mandibular lingual release, transpharyngeal, or transmandibular access—to ensure adequate exposure and complete resection with negative margins. Nonetheless, multiple studies have reported that concurrent CRT attains locoregional control and survival outcomes equivalent to those achieved with surgery, while offering the added advantage of preserving both anatomical integrity and functional capacity [16,17,18,19,20,21]. Following the publication of the GORTEC trial, CRT was adopted as the standard treatment for advanced-stage disease, irrespective of HPV status [3]. At the same time, the emergence of minimally invasive surgical approaches—such as TLM and TORS—has renewed interest in surgery as a primary modality, as these techniques allow for precise pathological staging and may enable adjuvant treatment de-intensification with reduced long-term morbidity [11,22].

In recent years, the epidemiology of OPSCC has shifted markedly, with HPV-associated disease now accounting for approximately 70% of new cases in many Western countries [23,24]. This HPV-related subset represents a distinct clinical entity characterized by improved prognosis and greater radiosensitivity, prompting efforts to de-escalate treatment to minimize long-term toxicity and preserve quality of life. Conversely, HPV-negative OPSCC—often associated with tobacco and alcohol use—continues to demonstrate poorer outcomes and remains underrepresented in current clinical trials. In regions with low HPV prevalence (<30%), such as parts of Asia, the applicability of these de-escalation protocols remains limited [25]. Furthermore, attempts to generate level I evidence for HPV-negative OPSCC, such as the RTOG 1221 trial, were terminated early due to poor accrual, leaving a gap in definitive guidance [26]. Against this backdrop, we conducted a matched-pair analysis comparing surgical and non-surgical approaches in HPV-negative OPSCC.

Despite therapeutic advances, survival for this subgroup remains unsatisfactory, with reported 5-year OS rates of approximately 50% in advanced stages [24]. Importantly, the superiority of RT/CRT observed in the overall matched cohort should not be extrapolated to all stages. In our stratified analysis, only stage III disease showed a statistically significant benefit in DSS, while early-stage and stage IV patients did not. This nuance is essential to avoid overgeneralizing the findings beyond the subgroup in which the effect was demonstrated. In our cohort, CRT achieved a 5-year OS of 59%, compared to only 28% for upfront surgery. Moreover, CRT was associated with significantly better DSS, particularly in stage III disease (87% vs. 42%; *p* = 0.01). These findings suggest that, in the absence of HPV-mediated radiosensitivity, CRT may offer superior outcomes to surgery, especially when extensive open approaches are required and they align with large-scale population-based analyses and bicentric matched-pair cohorts, which have shown no significant OS difference between surgery and CRT after adjustment for confounding variables, but highlight that over half of surgical patients ultimately receive trimodality therapy, increasing cumulative toxicity [13,24].

Previous studies comparing surgical and non-surgical approaches in advanced OPSCC have yielded inconsistent results [3,13,14,15,16,17,18]. While some have reported superior DSS with upfront surgery in stage III–IV disease [8,16,17,18,19,20,21,22], others, including Zenga et al. [27], found this advantage limited to HPV-positive cases. Matched-pair analyses from Kano et al. [16] and Zimmermann et al. [24] demonstrated equivalent survival for surgery and CRT in HPV-negative populations, with CRT associated with better long-term swallowing function. In contrast, our results showed poorer DSS in surgically treated patients, likely due to the absence of concomitant chemotherapy in adjuvant regimens—a limitation underscored by the landmark GORTEC trial, in which adding concurrent chemotherapy to RT significantly improved OS and locoregional control in advanced OPSCC [9].

Our results for non-surgical treatment (5-year OS of 59%) are consistent with contemporary series reporting 3-year OS rates ranging from 41% to 87% for CRT [7,28,29]. Meta-analyses suggest that surgery combined with CRT may yield the highest OS in some cohorts [9,15], but at the cost of increased morbidity. In our study, patients receiving CRT had significantly better metastasis-free survival and a lower incidence of second primary tumors, which may be attributable to the systemic effect of chemotherapy—a benefit absent in surgical monotherapy and often attenuated in adjuvant settings where chemotherapy is not indicated. The improved metastasis-free survival in the RT/CRT group should be interpreted cautiously, as most of these patients received concurrent chemotherapy, whereas the surgical cohort rarely received adjuvant systemic therapy. This treatment asymmetry likely contributed to the reduced metastatic risk and may partly explain the observed difference.

The role of induction chemotherapy (IC) in OPSCC remains uncertain. Randomized trials, such as the PARADIGM and DeCIDE studies, have not demonstrated an OS benefit from adding IC before CRT, although a potential benefit in patients at high risk of distant failure has been suggested [30,31]. However, concerns about toxicity were raised by the DeCIDE trial, which demonstrated a significant increase in serious adverse events in the induction therapy arm [32]. Future research should evaluate this strategy specifically in HPV-negative OPSCC, in which distant metastases remain a leading cause of treatment failure.

For early-stage OPSCC (e.g., cT1–T2, N0), single-modality treatment—either surgery or RT—achieves comparable oncologic outcomes, as confirmed in our series. In these cases, treatment choice is guided by patient preference, anticipated toxicity, and institutional expertise [24]. The superiority of one modality over the other has yet to be demonstrated, and ongoing randomized trials, such as EORTC 1420-HNCG-ROG (NCT02984410), aim to address this question by directly comparing state-of-the-art transoral surgery and RT in low-risk patients.

Toxicity and functional outcomes are central considerations in OPSCC management. Although our study does not include formal quality-of-life measurements, existing literature consistently reports that CRT is associated with long-term dysphagia, xerostomia, and speech impairment, whereas modern transoral surgical approaches may offer faster functional recovery and reduced late toxicity. These aspects critically influence treatment selection and reinforce that oncologic outcomes must be balanced against functional preservation. The absence of functional data in our cohort is therefore a relevant limitation that may affect interpretation.

This study presents several limitations. First, its retrospective design entails an inherent risk of selection bias. Although matching was performed to balance major prognostic variables (sex, subsite, T, N, and overall stage), unmeasured confounders may persist. Other relevant clinical determinants—such as age, alcohol and tobacco consumption, and comorbidity—were incorporated into the multivariable analysis, but the sample size limits its power to fully eliminate residual confounding. Consequently, the independent effect of treatment should be interpreted with caution. Second, quality-of-life and functional data were unavailable, preventing a comprehensive comparison of functional outcomes between treatment modalities. Third, the extended study period represents a major limitation. Surgery was performed between 1990 and 2009, whereas radiotherapy was delivered between 2009 and 2020. This temporal disparity encompasses substantial evolution in treatment protocols, both for surgery and for RT. Surgical patients treated in the earlier decades could not benefit from advances in transoral techniques, modern reconstructive options, improvements in anesthetic management, or optimized perioperative care that have been established over the past decade. At that time, small tumors were typically approached with minimally invasive surgery, whereas larger tumors underwent open resections without reconstruction. In contrast, current practice increasingly enables minimally invasive approaches combined with contemporary reconstruction, even in advanced cases, allowing R0 resections with improved functional preservation. These advances likely enhance oncologic and functional outcomes and would improve comparability with contemporary RT/CRT data. Similarly, treatment regimens for RT evolved substantially during the study window, and protocols were not fully standardized. Moreover, the fact that salvage surgery was used after RT failure introduces an additional source of bias. Another limitation concerns the adjuvant treatment profile within the surgical cohort. Only 66% of patients received postoperative radiotherapy, and none received adjuvant CRT, reflecting the protocols of the time. This likely contributed to differences in survival and metastatic control, particularly considering the well-established benefit of adjuvant CRT in locally advanced disease since the late 1990s. Finally, although data from other head and neck subsites suggest a potential benefit of surgery in selected scenarios, current evidence in OPSCC supports an individualized therapeutic approach, balancing oncologic control with functional preservation and minimization of toxicity.

## 5. Conclusions

In summary, our findings indicate that RT/CRT achieves survival outcomes comparable to surgery in HPV-negative OPSCC, particularly in advanced-stage disease, and may offer advantages in distant metastasis control and in reducing the incidence of second primary tumors. These data support the role of non-surgical organ-preserving approaches as a valid therapeutic option for HPV-negative OPSCC. However, these results should be interpreted within the broader context of current 2025 guidelines, which recognize multiple standard modalities for HPV-negative OPSCC. Treatment selection must therefore consider tumor stage, patient comorbidities, functional expectations, and local expertise. Notably, contemporary transoral surgical approaches combined with risk-adapted adjuvant RT achieve excellent oncologic outcomes, frequently exceeding 80% overall survival in appropriately selected patients. Consequently, our findings do not imply universal superiority of RT/CRT over surgery but rather highlight that non-surgical treatment constitutes an effective alternative, particularly in stage III disease.

## Figures and Tables

**Table 1:** Patient characteristics.

	All Patients N = 150 (%)	Radio (Chemo)-Therapy N = 50 (%)	Surgery Group N = 100 (%)	*p*
**Age**				0.32
<55 years	49 (33)	13 (26)	36 (36)	
55–59 years	26 (17)	11 (22)	15 (15)	
>59 years	75 (50)	26 (52)	49 (49)	
**Gender**				Matched
Female	0 (0)	0 (0)	0 (0)	
Male	150 (100)	50 (100)	100 (100)	
**Alcohol**				0.001
No	16 (11)	11 (22)	5 (5)	
Light drinker	13 (9)	9 (18)	4 (4)	
Moderate drinker	47 (31)	15 (30)	32 (32)	
Heavy drinker	74 (49)	15 (30)	59 (59)	
**Tobacco**				0.1
No	8 (5)	7 (14)	1 (1)	
Light smoker	65 (44)	18 (36)	47 (47)	
Moderate smoker	59 (39)	21 (42)	38 (38)	
Heavy smoker	18 (12)	4 (8)	14 (14)	
**Location**				Matched
Base of tongue	51 (34)	17 (34)	34 (34)	
Tonsil	99 (66)	33 (66)	66 (66)	
**T classification**				Matched
1	9 (9)	3 (6)	6 (6)	
2	39 (26)	13 26)	26 (26)	
3	54 (36)	18 (36)	36 (36)	
4a	48 (32)	16 (32)	32 (32)	
**N classification**				Matched
N0	51 (34)	17 (34)	34 (34)	
N1	21 (14)	7 (14)	14 (14)	
N2a	15 (10)	5 (10)	10 10)	
N2b	33 (22)	11 (22)	22 (22)	
N2c	27 (18)	9 (18)	18 (18)	
N3	3 (2)	1 (2)	3 (2)	
**Disease stage**				Matched
I	6 (4)	2 (4)	4 (4)	
II	24 (16)	8 (16)	16 (16)	
III	27 (18)	9 (18)	18 (18)	
IV	93 (62)	31 (62)	62 (62)	
**Histological grade**				0.01
G1	70 (47)	14 (28)	56 (56)	
G2	56 (37)	27 (54)	29 (29)	
G3	24 (16)	9 (18)	15 (15)	
**Follow-up mean (median), months**	49 (31)	47 (40)	50 (28)	0.8

Chi-squared test. *p* value comparing who received chemoradiotherapy vs. patients treated with surgery.

## Data Availability

The data presented in this study are under the custody of the Health Service of the Principality of Asturias and are therefore unavailable for sharing. The data are available on request from the corresponding author after an appropriate data sharing and access agreement is formally completed.
